# Elevated CO_2_ not increased temperature has specific effects on soil nematode community either with planting of transgenic *Bt* rice or non-*Bt* rice

**DOI:** 10.7717/peerj.8547

**Published:** 2020-02-12

**Authors:** Yingying Song, Jiawen Liu, Fajun Chen

**Affiliations:** Department of Entomology, College of Plant Protection, Nanjing Agricultural University, Nanjing, China

**Keywords:** Global climate change, Transgenic Bt crop, Soil fauna, Trophic group, Ecological index

## Abstract

**Background:**

Transgenic *Bt* rice has not been approved for commercial cultivation because of the fierce public debate on food safety, biosafety regulation and ecological risk. Meanwhile, the concentration of CO_2_ and temperature in the atmosphere, as important environmental factors affecting the persistence of exogenous* Bt* protein, have increased. Elevated CO_2_, increased temperature, the planting of transgenic *Bt* rice and their interactions may further influence the structure and complexity of soil food web. However, the effects of transgenic *Bt* rice planting on soil organism remain largely unexplored before its commercial production especially under global climate change.

**Methods:**

Here, we assessed the influences of transgenic *Bt* rice (cv. HH with fused *Cry1Ab*/*Cry1Ac* in contrast to its parental line of non-*Bt* rice cv. MH63) on soil nematode communities under the conditions of elevated CO_2_ concentration and increased temperature for 2 years of 2016 and 2017 in open-top chambers located in Ningjin County, Shandong Province of China.

**Results:**

Elevated CO_2_ concentration remarkably increased the abundance of fungivores and significantly decreased their nematode channel ratio (NCR) and enrichment index (*EI*) irrespective of rice variety (transgenic *Bt* rice or non-*Bt* rice) or temperature (normal temperature or increased temperature). Additionally, rice variety and temperature did not significantly change soil nematode composition, abundance and ecological indices (including total maturity index (∑*MI*), Shannon diversity (*H*′), structure index (*SI*), NCR and *EI*). However, apparent seasonal changes were observed in theses aforementioned variables.

**Discussion:**

These results suggested that atmospheric CO_2_ concentration but not temperature or rice variety has great impacts on soil nematode community, especially fungivores.

## Introduction

Transgenic *Bt* rice is genetically modified with the exogenetic gene of *Bacillus thuringiensis* (*Bt*) bacterium that codes for insecticidal proteins, and it has been used to improve crop yields and reduce the application of conventional insecticides (Chen, Shelton & Ye, 2011b). The China’s Ministry of Agriculture (CMOA) issued safety certificates for transgenic *Bt* rice lines of Huahui-1 (HH) and *Bt* Shanyou 63 (*Bt-* SY63) both expressing fused *Cry1Ab*/*Cry1Ac* proteins in 2009 (Jia, 2010) and 2014 (Li et al., 2016), while they have not yet been approved for commercial cultivation because of the fierce public debate on food safety, biosafety regulation and ecological risk (Mendelsohn et al., 2003). The exogenous *Bt* proteins from transgenic *Bt* crops can be released into soil through root exudates during whole growing season (Saxena, Flores & Stotzky, 1999), plant residues after havest of the crop (Stotzky, 2000) and pollen during tasseling (Losey, 1999), and thereby the toxin adsorbs and binds on surface-active particles in the soil (Saxena & Stotzky, 2001). Whether *Bt* proteins from transgenic *Bt* crops persist or accumulate in soil and then cause toxic effects on non-target soil organisms are serious problems. To date, the influences of transgenic *Bt* crops on soil ecosystem have been intensively studied (Romeis et al., 2019; Zuo et al., 2018; Chen et al., 2017a; Zhao et al., 2017); however, the persistence of exogenous *Bt* protein from transgenic *Bt* crops depends on many factors, such as temperature, soil types, pH and the type of *Bt* proteins (Xue, Diaz & Thies, 2014; Feng et al., 2011; Shen et al., 2008). Furthermore, there is still a limited number of researches on the importance of different environmental conditions on the ecological impacts of transgenic *Bt* crops.

The global atmospheric carbon dioxide (i.e., CO_2_) concentration has increased rapidly since the industrial revolution, because of increased human activities and rapid economic development (IPCC, 2018). It has reached 415.26 ppm in 2019 (Mauna Loa Observatory: NOAA-ESRL), and is predicted to 430-1000 ppm by the end of this century (IPCC, 2014). Meanwhile, the increasing emission of CO_2_ will lead to an increase of global mean surface air temperature about 1.5 °C by 2100 (IPCC, 2018). As important environmental factors affecting plant performance worldwide, especially the C_3_ crops such as cotton and rice (Ainsworth & Long, 2005; Chen, Ge & Parajulee, 2005), the interactions between elevated CO_2_ and increased temperature can influence plant growth (Tan et al., 2018; Xu et al., 2016), enhance carbon allocation belowground (Hu et al., 2017; Ainsworth & Long, 2005), change the composition and amount of root exudation and decrease the pH of rhizosphere soil (Zhao et al., 2016). Furthermore, these variations may in turn affect soil fauna indirectly, and furtherly influence the structure and complexity of soil food web (Hu et al., 2017; García-Palacios et al., 2015). Sun et al. (2010) found that elevated CO_2_ changed the interactions between nematode and tomato genotypes. García-Palacios et al. (2015) reported that global change have significantly effects on soil microbes and ecosystem functioning. Therefore, it is necessary and important to study the impacts of elevated CO_2_ and increased temperature on soil ecosystem.

Soil nematodes, one of the most abundant invertebrates, occupy several trophic levels in the food webs and exhibit differences in life history strategies (Bongers & Ferris, 1999). Besides, they also play significant roles in nutrient cycling and response to variations in environmental conditions (Neher, 2010). Due to their sensitivity to physical and chemical disturbances, soil nematodes showed a great potential in the assessment of environmental risk and ecosystem health. Several studies have reported that the effects of transgenic *Bt* crops on nematode communities. For example, Čerevková et al. (2018) reported that soil nematode communities were not significantly influenced by transgenic *Bt* maize planting. Liu et al. (2015) monitored the changes of soil nematode and microbial communities to transgenic *Bt* oilseed rape, and there were no significant differences. Chen et al. (2017a) found that the planting of transgenic *Bt* rice reduced phytoparasitic nematode abundance. However, most of the studies did not take the interactions between transgenic crops and future climate changes into account simultaneously.

In order to evaluate the impacts of transgenic *Bt* rice planting on soil ecosystem under global climate change, we analyzed the community composition, abundance and ecological indices of nematodes in soils planted with transgenic *Bt* rice expressing fused *Cry1Ab/Cry1Ac* proteins (cv. HH) and its near-isogenic parent line (cv. MH) under the elevated CO_2_ and temperature conditions. We hypothesized that (i) soil nematodes would not respond to transgenic *Bt* rice cultivation under ambient atmospheric CO_2_ concentration or temperature, (ii) nematode community composition would also be similar between transgenic *Bt* rice and the parent line of non-*Bt* rice under elevated CO_2_ concentration because this condition adversely influenced *Bt*-gene expression for the transgenic *Bt* rice (Chen et al., 2011a), but (iii) herbivorous nematodes would be decreased under elevated CO_2_ concentration via reduces in food quality (the higher ratio of organic carbon to nitrogen) for herbivorous nematodes (Hu et al., 2017).

## Materials and Methods

### Open-top chambers(OTCs)

The experiment was conducted in twelve open-top chambers (2.5 m in height and 4.2 m in diameter) at the Innovation Research Platforms for Climate Change, Biodiversity and Pest Management (CCBPM; http://www.ccbpm.org) in 2016 and 2017, which is located in Ningjin County, Dezhou City, Shandong Province of China (37.64°N, 116.8°E). This region has a warm and semi-humid monsoon climate, with an annual mean temperature of 12.9 °C and annual mean precipitation of 547.5 mm. The CO_2_ concentrations were continuously monitored and adjusted with an infrared CO_2_ analyzer (Ven-tostat 8102, Telaire Company, Goleta, CA, USA) at an interval of 20 min every day, and the temperature was measured three times a day using an automatic temperature analysis system (U23-001, HOBO Pro V2 Temp/RH Data Logger; MicroDAQ Ltd, Contoocook, NH; The accuracy of temperature was defined as ± 0.02 °C from 0 to 50 °C). Two levels of CO_2_ concentration, including the ambient (A, 382 ± 4.02 µl/L) and the elevated (E, 754 ± 3.70 µl/L) levels, and two temperature levels, including the normal (25.57 ± 0.41 °C) and the increased (26.14 ± 0.43  °C) levels were applied continuously in the OTCs. Three blocks were used for CO_2_ and temperature treatments and each block was split into four OTCs, i.e., one with ambient CO_2_ concentration and increased temperature (A+T), one with ambient CO_2_ concentration and normal temperature (A-T), one with elevated CO_2_ concentration and increased temperature (E+T), and one with elevated CO_2_ concentration and normal temperature (E-T).

### Rice cultivars and planting

Transgenic *Bt* rice (cv. HH with fused *Cry1Ab*/*Cry1Ac*) and its near-isogenic parent line of non-*Bt* rice (cv. MH) were both provided by the College of Plant Science and Technology of Huazhong Agricultural University, Wuhan City, Hubei Province of China. These two cultivars of rice were planted in the plastic pots (32 cm in height and 24 cm in diameter; containing 10 kg sifted field soil) located in the OTCs on June 11 in 2016 and 2017 respectively. Thirty pots for each cultivar were placed randomly in each OTC. The potted field soil was collected from sifted field soil. The fields have been planted with conventional crops, such as corn, wheat, cotton, but transgenic crops had never previously been cultivated within 100 km. The chemical properties were as follows: pH 8.5, organic C 6.6 g kg^−1^, total N 0.4 g kg^−1^, alkaline hydrolysis N 7.9 g kg^−1^, available P 14.4 mg kg^−1^ and available K 96 mg kg^−1^. The soil belong to fluvo-aquic soil with sandy texture. After growing for 30 days, the rice seedlings were thinning to twelve plants in each pot. To minimize the effect of microclimate, the pots were weekly rotated within the chambers. Normal cultural practices for rice cultivation, such as fertilization and irrigation, were followed except that no insecticides were applied during the entire experimental periods.

### Soil sampling

Soil samples were collected in June (seedling stage) and October (harvesting stage) of 2016 and 2017 respectively, and in August (shooting stage) of 2017. Five sampling pots for each cultivar in each block were randomly selected. In each pot, four soil cores (2.5 cm in diameter) from 0 to 10 cm surface soil were randomly collected, and they were mixed together as a composite sample, and then kept in 4 °C for less than a week until identification. Before nematode extraction, plant tissues including root fragments were removed from the soil samples using a two mm mesh sieve.

Nematodes were extracted from 100 g soils using the minor modified Baermann method (Liu et al., 2008). All individuals of sampled nematodes were counted under the stereoscopic microscope (XTL-BM-7B), and about 100–150 specimens per soil sample were randomly selected, then identified to genera and assigned to four trophic groups: (1) herbivore; (2) bacterivore; (3) fungivore; (4) omnivore-predator (Yeates et al., 1993).

### Nematode ecological index

The total number of nematodes at each sampling time was used as an index of abundance expressed as individuals per 100 g dry soil (Liu et al., 2018). Besides, the relative abundance of the dominant, common and rare groups were >10%, ≤10%, and <1% of the total nematodes, respectively (Liang, Zhang & Li, 2001). The ecological indices of nematode were evaluated by total maturity index (∑*MI*; a measure of disturbance), Shannon diversity (*H*′: an indicator of diversity index), nematode channel ratio (NCR: an indicator of the prevalence of organic matter decomposition), enrichment index (*EI*: an indicator of soil food web condition) and structure index (*SI*: a measure of food web length and connectance). The above indices were calculated using the following equations:

(1) \begin{eqnarray*}\text{Total maturity index}(\sum MI):\sum MI=\sum {v}_{i}{f}_{i}\end{eqnarray*}

(2) \begin{eqnarray*}\text{Shannon diversity}({H}^{{^{\prime}}}):{H}^{{^{\prime}}}=-\sum {P}_{i}\ln \nolimits {P}_{i}\end{eqnarray*}

(3) \begin{eqnarray*}\text{Nematode channel ratio (NCR)}:\mathrm{NCR}\,=\,\,\mathrm{Ba}/(\,\mathrm{Ba}+\,\mathrm{Fu})\end{eqnarray*}

(4) \begin{eqnarray*}\text{Enrichment index}(EI):EI=100\times (e/(e+b))\end{eqnarray*}

(5) \begin{eqnarray*}\text{Structure index}(SI):SI=100\times (s/(s+b))\end{eqnarray*}

Where, *v*_*i*_ was given c-p value based on life history strategies of free life and plant parasitic nematodes in ecological succession, *f*_*i*_ represents one genus proportion in total nematodes (Bongers, 1990); *P*_*i*_ is the proportion of individuals belongs to the ith taxon in the total number of individuals of nematode (Shannon & Weaver, 1949); Ba represents bacterivores and Fu represents fungivores; *e* (enrichment component), *b* (basal component) and *s* (structural component) are calculated using those guilds indicating enrichment (Ba_1_, Fu_2_), basal (Ba_2_, Fu_2_) and structure (Ba_3_-Ba_5_, Fu_3_-Fu_5_, Om_3_-Om_5_, Ca_2_-Ca_5_), respectively (Ferris, Bongers & De Goede, 2001).

### Data analysis

All statistical analyses were performed with R software version R 3.0.3. Prior to statistical analysis, the Shapiro–Wilk and Levene’s tests were applied to evaluate data normality and homogeneity respectively. Nematode abundance was log(x+1) transformed and the percentage of nematode were arcsine square-root transformed for further statistical analysis, but untransformed means were presented in figures and tables. Variables were evaluated by use of split-plot analysis of variance for the repeated-measures analysis to measure the effects of rice variety (HH vs. MH), CO_2_ (382 µl/L vs. 754 µl/L), temperature (25.57 °C vs. 26.14 °C) and sampling time (Jun and Oct in 2016, and Jun, Aug and Oct in 2017) and their interactions on the nematode community. The CO_2_ and temperatue were assigned as main treatments, and variety of rice was assigned as a split-plot in the split-plot design. When there are interactive effects between sampling time and variety, then we compared the main and interactive treatment effects within each sampling time. Moreover, if the main effects and their interactions with different sampling season were significant, then we do one-way ANOVA to test the differences in these above parameters on each sampling date. The LSD test was used to analyze the significant differences between treatments at *P* <0.05. Non-metric multidimensional scaling (NMDS) was performed to determine which factors (rice variety, CO_2_ concentration, temperature and sampling time) were markedly correlated with the NMDS ordination of soil nematode community by returning squared correlation coefficients (i.e., envfit function, Vegan package in R) and the Bray-Curtis distance analysis was used to evaluate the dissimilarity of nematode community composition across rice variety, CO_2_ concentration, temperature and sampling time.

## Results

### Community composition and dissimilarity comparison

**Figure 1:**
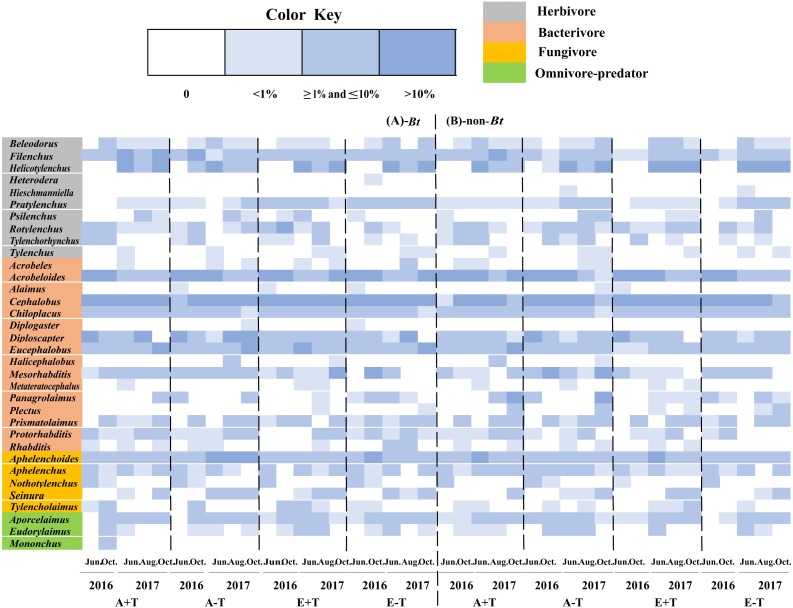
Community composition and the relative abundance of soil nematode in the fields of transgenic *Bt* rice and parental line of non-*Bt* rice grown under different CO_2_ concentrations and temperature. (A) Fields of transgenic *Bt* rice; (B) fields of non-*Bt* rice; >10%-dominant group of soil nematode; ≥1% and ≤10%—common group of soil nematode; <1%—rare group of soil nematode; A + T-indicated ambient CO_2_ concentration and increased temperature; A–T-indicated ambient CO_2_ concentration and normal temperature; E + T-indicated elevated CO_2_ concentration and increased temperature; E − T—indicated elevated CO_2_ concentration and normal temperature; the same as in the following figures.

A total of 34 genera of nematode were observed in the sampled soil collected during two consecutive years of 2016 and 2017. There was no difference in the nematode variables between transgenic *Bt* rice (cv. HH) and the parental line of non-*Bt* rice (cv. MH) in all the CO_2_ and temperature conditions (i.e., A+T, A-T, E+T, E-T), except that *Heterodera*, *Diplogaster* and *Mononchus* were just found in the soil of transgenic *Bt* rice (cv. HH), and *Hieschmanniella* was only observed in the soil of non-*Bt* rice (cv. MH) (Fig. 1).

**Figure 2:**
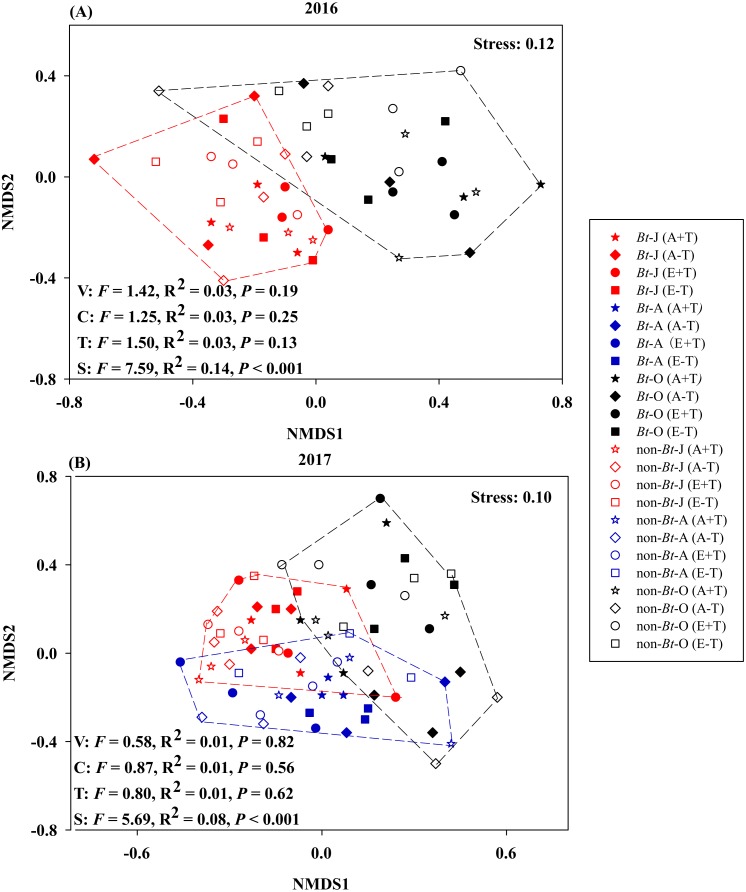
Non-metric multidimensional scaling of sampling time, rice variety, CO_2_ concentration and temperature on the community composition of nematode in the soil of transgenic *Bt* rice and non-*Bt* rice. (A) 2016; (B) 2017; NMDS - non-metric multidimensional scaling; S, sampling time; V, rice variety; C, CO_2_ concentration; T, temperature; -J, June; -A, August; -O, October.

The results of NMDS consistently showed that variety, CO_2_ concentration and temperature have no significant effects on nematode communities during 2016–2017 (Fig. 2).

### Abundance

**Figure 3:**
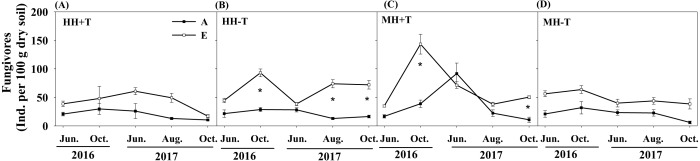
The absolute abundance of soil fungivores in the soil of transgenic *Bt* rice and non-*Bt* rice grown under different CO_2_ concentrations and temperature. (A) HH + T, transgenic *Bt* rice under the condition of increased temperature; (B) HH-T, transgenic *Bt* rice under the condition of normal temperature; (C) MH + T, the parental line of non-*Bt* rice under the condition of increased temperature; (D) MH-T, the parental line of non-*Bt* rice under the condition of normal temperature; A, the condition of ambient CO_2_ concentration; E, the condition of elevated CO_2_ concentration; * indicated significant difference among the conditions of CO_2_ concentration by the Fisher’s LSD test at *P* = 0.05.

The treatments of rice variety, CO_2_ concentration or temperature didn’t significantly affect the total nematode abundance (Table 1). Although the absolute abundances of four trophic groups were not significantly influenced by rice variety or temperature, the absolute abundance of fungivores in the condition of elevated CO_2_ concentration was remarkably different from that in the condition of ambient CO_2_ concentration. One-way ANOVA further showed that the absolute abundance of fungivores was significantly higher in the condition of elevated CO_2_ concentration than that in the condition of ambient CO_2_ concentration for HH-T treatment (i.e., transgenic *Bt* rice under the condition of normal temperature) in October of 2016 and 2017 and in August of 2017, and for MH+T treatment (i.e., the parental line of non-*Bt* rice under the condition of increased temperature) in October of 2016 and 2017 (*P* < 0.05; Figs. 3B–3C).

**Table 1:** The effects of rice variety (transgenic *Bt* rice vs. its parental line of non-*Bt* rice), CO_2_ concentration (ambient CO_2_ concentration vs. elevated CO_2_ concentration), temperature (normal temperature vs. increased temperature), sampling time (Jun. and Oct. in 2016, and Jun. Aug. and Oct. in 2017) and their interactions on nematode abundance and ecological indices (*F* value).

Variables	Abundance					Ecological indices				
	Total	Herbivores	Bacterivores	Fungivores	Omnivores- predators	Σ*MI*	*H’*	NCR	*EI*	*SI*
Variety (V)	3.30	0.13	1.22	0.73	0.46	0.15	1.99	0.00	0.23	0.58
CO_2_ (C)	0.22	0.87	0.31	6.52^*^	0.35	3.80	1.93	4.60^*^	5.37^*^	1.22
Temperature (T)	0.01	0.32	0.17	0.51	0.16	1.32	0.45	0.003	1.44	1.05
Sampling time (S)	16.12^***^	56.19^***^	23.27^***^	5.42^*^	26.05^***^	29.57^***^	41.15^***^	17.68^***^	15.53^***^	26.71^***^
V × C	0.28	0.89	0.72	0.43	0.17	0.21	0.46	0.64	0.43	0.24
V × T	2.40	2.51	0.65	2.97	0.65	0.92	0.78	1.14	0.52	1.03
V × S	3.05	1.67	0.58	0.19	0.23	3.10	9.25^**^	4.30^*^	1.68	0.45
C × T	0.09	1.73	0.64	0.36	0.55	0.46	0.00	0.17	0.53	1.56
C × S	1.65	2.05	1.13	0.53	0.08	4.04	0.01	0.02	0.62	2.62
T × S	0.21	0.04	0.22	1.65	0.16	2.38	0.11	0.16	1.37	0.19
V × C × T	0.94	1.36	0.71	1.90	0.49	0.01	0.01	0.21	1.99	0.58
V × C × S	1.25	2.11	3.20	0.86	0.68	3.15	2.08	3.24	0.90	2.80
V × T × S	0.34	1.47	0.83	0.77	0.33	0.43	0.57	0.29	0.35	0.36
C × T × S	2.60	2.04	1.09	0.84	1.97	0.002	0.19	0.74	1.80	2.68
V × C × T × S	0.03	3.07	1.95	2.01	0.59	0.24	0.11	0.47	0.48	1.97

**Notes.**

Σ*MI* total maturity index *H*′ Shannon diversity NCR nematode channel ratio *EI* enrichment index *SI* structure index

Sampling time was assigned as repeated factor.

* *P* < 0.05.

** *P* < 0.01.

*** *P* < 0.001.

### Ecological indices

**Figure 4:**
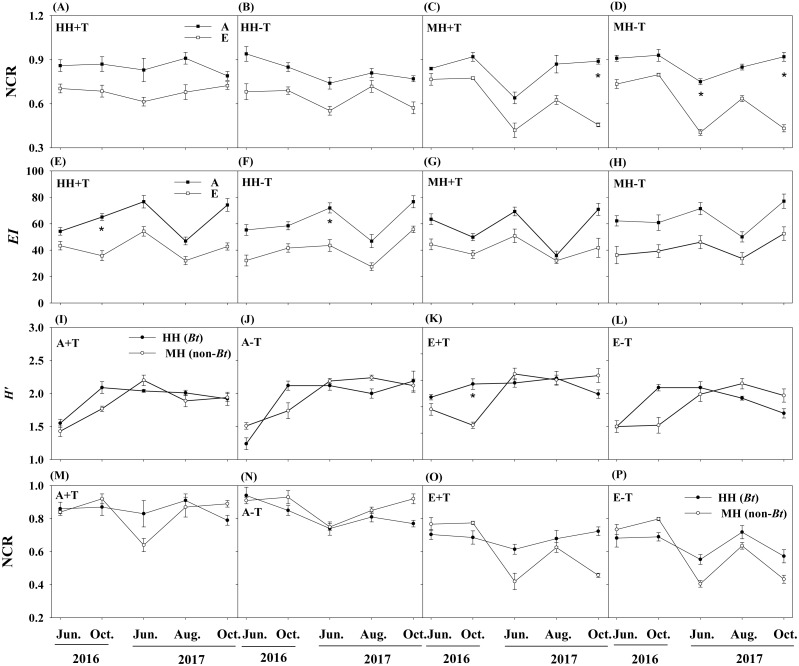
Ecological indices of NCR, *EI* and *H*′ in the soil of transgenic *Bt* rice and non-*Bt* rice grown under different CO_2_ concentrations and temperature. (A–D) Nematode channel ratio (i.e., NCR) between different conditions of CO_2_ concentration; (E–H) enrichment index (i.e., *EI*) between different conditions of CO_2_ concentration; (I-L) Shannon diversity (i.e., *H*′) between transgenic *Bt* rice and non-*Bt* rice; (M-P) nematode channel ratio (i.e., NCR) between transgenic *Bt* rice and non-*Bt* rice; HH+T - transgenic *Bt* rice under the condition of increased temperature; HH-T, transgenic *Bt* rice under the condition of normal temperature; MH + T, the parental line of non-*Bt* rice under the condition of increased temperature; MH − T, the parental line of non-*Bt* rice under the condition of normal temperature; A + T, ambient CO_2_ concentration and increased temperature; A − T, ambient CO_2_ concentration and normal temperature; E + T, elevated CO_2_ concentration and increased temperature; E − T, elevated CO_2_ concentration and normal temperature; A, the condition of ambient CO_2_ concentration; E, the condition of elevated CO_2_ concentration; * indicated significant difference by the Fisher’s LSD test at *P* = 0.05.

**Figure 5:**
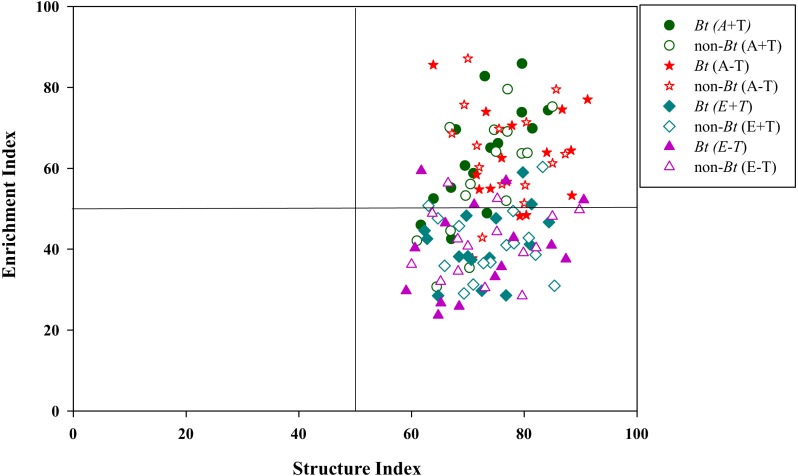
Nematode faunal analysis in the soil of transgenic *Bt* rice and non-*Bt* rice grown under different CO_2_ concentrations and temperature.

There were no pronounced changes in the measured ecological indices of soil nematodes between rice variety treatments or temperature treatments (Table 1). However, elevated CO_2_ concentration significantly influenced the ecological indices of NCR and *EI*. One-way ANOVA also showed that the ecological index of NCR was significantly lower in the condition of elevated CO_2_ concentration than that in the condition of ambient CO_2_ concentration for MH+T treatment in October of 2017, and for MH-T treatment (i.e., the parental line of non-*Bt* rice under the condition of normal temperature) in June and October of 2017 (*P* < 0.05; Figs. 4C–4D). The ecological index of *EI* was significantly lower in the condition of elevated CO_2_ concentration than that in the condition of ambient CO_2_ concentration for HH+T treatment (i.e., transgenic *Bt* rice under the condition of increased temperature) in October of 2016, and for HH-T treatment in June of 2017 (*P* < 0.05; Figs. 4E–4F). Besides, significant interaction between rice variety and sampling time on *H′* and NCR were observed. Nevertheless, the treatment of rice variety or CO_2_ or temperature or their interaction did not significantly influence the ecological indices of *H′* and NCR within each sampling time (Table 2). One-way ANOVA further showed that the ecological indices of *H′* and NCR were not significantly different between rice variety treatments within each sampling time except that in October of 2016 (Fig. 4). The split-plot analysis of variance and the nematode faunal analysis also showed markedly fluctuations among sampling time (Table 1; Fig. 5).

## Discussion

### Rice cultivar effects on soil nematode

Our findings confirmed that transgenic *Bt* rice (cv. HH) didn’t remarkably change nematode community abundance and ecological indices under ambient CO_2_ and temperature conditions. These results lead us to support the hypothesis (i) and these were consistent with previous studies that transgenic *Bt* plants have no detrimental effects on non-target soil fauna under ambient CO_2_ concentration and temperature conditions (Romeis et al., 2019; Liu et al., 2018; Zhao et al., 2017). The *Bt* protein content was relatively low in soil (Chen et al., 2017b; Liu et al., 2017) and degraded rapidly in soils (Liu et al., 2018; Valldor et al., 2015), these findings may be likely responsible for above results. To our knowledge, few studies have reported the influences of transgenic *Bt* crops on non-target soil organisms under elevated CO_2_ concentration and temperature, which may indirectly change the structure, activities and ecosystem functions of the soil biota, as well as the soil food webs (García-Palacios et al., 2015; Blankinship, Niklaus & Hungate, 2011). In the current study, we found that there were no significant differences between rice variety on nematode community composition under elevated CO_2_ concentration or elevated temperature condition, which lead us to support the hypothesis (ii). We believed that the effects of transgenic *Bt* rice on soil nematode would not be influenced by global change.

**Table 2:** The effects of rice variety (transgenic *Bt* rice vs. its parental line of non-*Bt* rice), CO_2_ concentration (ambient CO_2_ concentration vs. elevated CO_2_ concentration), temperature (normal temperature vs. increased temperature) and their interactions on nematode ecological indices of *H′* and NCR within each sampling time (*F* value).

Variables	Jun. 2016		Oct.2016		Jun.2017		Aug. 2017		Oct.2017	
	*H′*	NCR	*H′*	NCR	*H′*	NCR	*H′*	NCR	*H′*	NCR
Variety (V)	0.04	0.15	0.78	3.49	0.20	4.41	1.12	0.11	1.00	0.05
CO_2_ (C)	0.96	0.10	4.11	0.17	0.38	0.09	0.00	0.90	1.36	0.76
Temperature (T)	0.76	0.68	1.19	0.01	0.01	0.33	1.59	0.39	0.24	0.26
V × C	0.81	2.06	1.76	0.24	0.08	0.03	0.05	0.07	1.33	3.55
V × T	2.25	0.13	0.10	0.50	0.30	1.84	4.36	0.43	0.07	0.04
C × T	0.06	4.05	1.25	0.04	0.01	0.13	1.20	2.31	1.16	0.35
V × C × T	0.17	0.06	0.33	0.01	0.08	0.61	0.17	0.41	0.05	0.04

**Notes.**

*H*′ Shannon diversity NCR nematode channel ratio

### Global change effects on soil nematode

Although elevated CO_2_ concentration did not change the absolute abundance of herbivores, this condition remarkably increased the absolute abundance of fungivores. Thus, we can reject the hypothesis (iii). Additionally, the rising atmospheric CO_2_ concentration often increases the rice root biomass (Hu et al., 2017; Yang et al., 2010) by enhancing carbon allocation belowground (Ainsworth & Long, 2005). Moreover, García-Palacios et al. (2015) reported that soil fungal abundance responding to elevated CO_2_ concentration was positively correlated with plant biomass. Therefore, we speculated that a series of above variations may in turn indirectly affect the abundance of soil fungivores under elevated CO _2_condition. Hu et al. (2017) found that elevated CO_2_ concentration remarkably increased the abundance of herbivores for the rice cultivar of IIYou084 and significantly reduced the the abundance of herbivores for the rice cultivar of Wuyuniing at the ripening stage. Thus, we assumed that the effect of elevated CO_2_ concentration on the abundance of trophic groups was cultivar-specific.

The ecological indices were essential measures of indicating soil health conditions (Chen et al., 2017a; Ferris et al., 2012; Li et al., 2005). High values of NCR (>0.5) were found in all treatments of A+T, A-T, E+T and E-T, suggesting that bacterivores were dominant in organic matter decomposition for both transgenic *Bt* rice (cv. HH) and its parental line of non-*Bt* rice (cv. MH). Interestingly, regardless of rice variety or temperature, the ecological index of NCR was pronouncedly declined under the elevated CO_2_ concentration, indicating that fungivores became increasingly important in degrading organic matter with the increase of CO_2_ concentration. In the present study, we also found that the elevated CO_2_ concentration remarkably decreased the ecological index of *EI*, and nematode faunal distributions under ambient CO_2_ concentration were the largest in quadrant B, while those under elevated CO_2_ concentration were primarily in quadrant C, irrespective of rice variety. Ferris, Bongers & De Goede (2001) inferred that the value of C/N ratio of the organic material in soil was higher in quadrant C than that in quadrant B according to nematode faunal analysis, and Sun et al. (2010) found that elevated CO_2_ concentration could increase C/N ratio in plant tissues. Thus, we speculated that the impact of CO_2_ concentration on nematode faunal distribution may be related to increased C/N ratio in plant tissues.

### Dynamics of nematodes in controlled environment

The current study was conducted in open-top chambers, where the CO_2_ concentration and temperature were tightly controlled. The experiment eliminated the influence of environmental variations present in realistic environment. However, sampling time still significantly changed the nematode composition, abundance and ecological indices. There were several reasons could interpret the fact of temporal dynamics in planted rice soil. First, the wetting-drying cycles caused by irrigation may alter the biological and biochemical activity (Chen et al., 2017b), and indirectly changed the nematode assemblage. Second, crop phenology may affect the population of soil nematode, and this finding agreed with data from previous reports on rhizospheric methanotroph community (Vishwakarma et al., 2009) and fungus-nematode (Bloomberg & Sutherland, 2010).

## Conclusions

In this study, we tried to evaluate the impacts of transgenic *Bt* rice on soil ecosystem in the simulated condition of global climate change, since the majority of studies focused on ambient CO_2_ concentration and normal temperature. Our results showed that transgenic *Bt* rice has negligible influence on soil nematode community in elevated CO_2_ concentration and temperature. However, only one transgenic *Bt* rice (cv. HH), expressing fused *Cry1Ab*/*Cry1Ac* proteins, was assessed in the present study. Given the potential impacts of crop cultivars, *Bt* transgenic events and environmental factors on the exogenous *Bt* expression and persistence, some long-term studies addressing the ecological safety of transgenic *Bt* rice under the conditions of rising CO_2_ concentration and temperature are still necessary to carry out in the future.

##  Supplemental Information

10.7717/peerj.8547/supp-1 Data S1 Raw Data

## Additional Information and Declarations

### Data Availability

The following information was supplied regarding data availability: The raw data are available in a Supplemental File.
